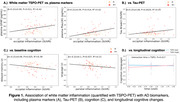# Inflammation in the white matter relates to core Alzheimer's disease pathophysiological processes

**DOI:** 10.1002/alz.093072

**Published:** 2025-01-09

**Authors:** Julie Ottoy, Min Su Kang, Eric Yin, Nesrine Rahmouni, Jenna Stevenson, Jean‐Paul Soucy, Andrea L. Benedet, Kaj Blennow, Henrik Zetterberg, Nicholas J. Ashton, Serge Gauthier, Sandra E Black, Pedro Rosa‐Neto, Maged Goubran

**Affiliations:** ^1^ LC Campbell Cognitive Neurology Research Unit, Sunnybrook Research Institute, University of Toronto, Toronto, ON Canada; ^2^ Translational Neuroimaging Laboratory, The McGill University Research Centre for Studies in Aging, Montréal, QC Canada; ^3^ McConnell Brain Imaging Centre ‐ McGill University, Montreal, QC Canada; ^4^ Department of Psychiatry and Neurochemistry, Institute of Neuroscience and Physiology, The Sahlgrenska Academy, University of Gothenburg, Mölndal, Gothenburg Sweden; ^5^ Department of Psychiatry and Neurochemistry, Institute of Neuroscience and Physiology, The Sahlgrenska Academy, University of Gothenburg, Mölndal Sweden; ^6^ Clinical Neurochemistry Laboratory, Sahlgrenska University Hospital, Mölndal Sweden; ^7^ Hong Kong Center for Neurodegenerative Diseases, Hong Kong China; ^8^ Department of Neurodegenerative Disease, UCL Institute of Neurology, London UK; ^9^ UK Dementia Research Institute, University College London, London UK; ^10^ King’s College London, Institute of Psychiatry, Psychology & Neuroscience, Maurice Wohl Clinical Neuroscience Institute, London UK; ^11^ NIHR Biomedical Research Centre for Mental Health & Biomedical Research Unit for Dementia at South London & Maudsley NHS Foundation, London UK; ^12^ Translational Neuroimaging Laboratory, The McGill University Research Centre for Studies in Aging, Montreal, QC Canada; ^13^ Division of Neurology, Department of Medicine, University of Toronto, Toronto, ON Canada; ^14^ Montreal Neurological Institute, Montréal, QC Canada; ^15^ Physical Sciences Platform, Sunnybrook Research Institute, University of Toronto, Toronto, ON Canada; ^16^ Department of Medical Biophysics, University of Toronto, Toronto, ON Canada

## Abstract

**Background:**

In‐vivo PET imaging studies have demonstrated neuroinflammation (microglia reactivity) in the neocortex of Alzheimer’s disease (AD) patients. However, the extent and implication of microglia reactivity in white matter regions remains unclear. Here, we explored microglia reactivity in white matter using PET imaging of the translocator protein (TSPO) in relation to core AD biomarkers (amyloid, tau, and astrogliosis), microstructural damage, and cognitive decline.

**Method:**

Ninety‐one participants from the Translational Biomarkers in Aging and Dementia cohort (45% Aβ‐positive, 39% cognitively impaired) underwent diffusion‐weighted MRI, TSPO‐PET (^11^C‐PBR28), amyloid‐PET (^18^F‐NAV4694), tau‐PET (^18^F‐MK6240), and plasma Aβ42/40, ptau181, ptau217, and ptau231. We investigated the associations of TSPO‐PET SUVR in white matter lobes (eroded by 2mm^3^) with each of the AD biomarkers, diffusion metrics, and cognition, adjusted for demographics and global cortical TSPO‐PET. Longitudinally, we investigated the interaction between time (up to 2y) and baseline white matter TSPO‐PET SUVR on cognitive decline, adjusted for demographics and cortical amyloid.

**Result:**

White matter TSPO‐PET was increased globally with older age and frontally with male sex (p<0.05). In impaired individuals, TSPO‐PET was elevated in temporo‐occipital white matter regions compared to controls (p=0.01‐0.02). The occipital increases were associated with reactive astrogliosis (p=0.011) and higher‐Braak tau‐PET (p=0.003‐0.034 for Braak3‐4) and plasma ptau181 (p=0.014) (Figure 1A‐B), but not with Aβ, ptau231 or ptau217. In addition, higher occipital TSPO‐PET was associated with lower fibre integrity (fractional anisotropy, p=0.025) but not free water. Finally, in relation to cognition, higher occipital and parietal TSPO‐PET was significantly associated with impaired memory and language, respectively, independent of Aβ (Figure 1C). Longitudinally, within the same patient, higher white matter TSPO‐PET at baseline was significantly associated with favorable clinical outcome over time (Figure 1D).

**Conclusion:**

Increased microglia reactivity (TSPO‐PET) in posterior white matter may indicate later‐stage AD associated with astrogliosis, tau, and dysfunction of the memory and language domains. Another novel finding is that individuals with higher white matter TSPO‐PET at baseline displayed better clinical prognosis over time. As such, assessing white matter inflammation could be promising to evaluate target engagement in clinical trials of anti‐inflammatory drugs and to subtype patients for better patient‐centric treatment outcome.